# A Video Game Promoting Cancer Risk Perception and Information Seeking Behavior Among Young-Adult College Students: A Randomized Controlled Trial

**DOI:** 10.2196/games.5793

**Published:** 2016-07-28

**Authors:** Georges Elias Khalil, Ivan L Beale, Minxing Chen, Alexander V Prokhorov

**Affiliations:** ^1^ The M.D. Anderson Cancer Center Department of Behavioral Science University of Texas Houston, TX United States; ^2^ School of Psychology University of New South Wales New South Wales Australia; ^3^ The M.D. Anderson Cancer Center Division of Quantitative Sciences University of Texas Houston, TX United States

**Keywords:** cancer, risk, information seeking, perceived risk, perceived susceptibility, perceived severity, cancer prevention, games for health, serious games

## Abstract

**Background:**

Risky behaviors tend to increase drastically during the transition into young adulthood. This increase may ultimately facilitate the initiation of carcinogenic processes at a young age, highlighting a serious public health problem. By promoting information seeking behavior (ISB), young adults may become aware of cancer risks and potentially take preventive measures.

**Objective:**

Based on the protection motivation theory, the current study seeks to evaluate the impact of challenge in a fully automated video game called *Re-Mission* on young adult college students' tendency to perceive the severity of cancer, feel susceptible to cancer, and engage in ISB.

**Methods:**

A total of 216 young adults were recruited from a university campus, consented, screened, and randomized in a single-blinded format to 1 of 3 conditions: an intervention group playing *Re-Mission* at high challenge (HC; n=85), an intervention group playing *Re-Mission* at low challenge (LC; n=81), and a control group with no challenge (NC; presented with illustrated pictures of *Re-Mission*; n=50). Measurement was conducted at baseline, immediate posttest, 10-day follow-up, and 20-day follow-up. Repeated-measures mixed-effect models were conducted for data analysis of the main outcomes.

**Results:**

A total of 101 young adults continued until 20-day follow-up. Mixed-effect models showed that participants in the HC and LC groups were more likely to increase in perceived susceptibility to cancer (*P*=.03), perceived severity of cancer (*P*=.02), and ISB (*P*=.01) than participants in the NC group. The LC group took until 10-day follow-up to show increase in perceived susceptibility (B=0.47, standard error (SE) 0.16, *P*=.005). The HC group showed an immediate increase in perceived susceptibility at posttest (B=0.43, SE 0.14, *P*=.002). The LC group exhibited no changes in perceived severity (B=0.40, SE 0.33, *P*=.24). On the other hand, the HC group showed a significant increase from baseline to posttest (B=0.39, SE 0.14, *P*=.005), maintaining this increase until 20-day follow-up (B=−0.007, SE 0.26, *P*=.98). Further analyses indicated that perceived threat from virtual cancer cells in the game is related to the increase in perceived severity (B=0.1, SE 0.03, *P*=.001), and perceived susceptibility is related to changes in ISB at 10-day follow-up (B=0.21, SE 0.08, *P*=.008).

**Conclusions:**

The feature of challenge with cancer cells in a virtual environment has the potential to increase cancer risk perception and ISB. The results are promising considering that the *Re-Mission* intervention was neither designed for cancer risk communication, nor applied among healthy individuals. Further research is needed to understand the theoretical framework underlying the effects of *Re-Mission* on ISB. The findings call for the development of a Web-based, game-based intervention for cancer risk communication and information seeking among young adults.

**ClinicalTrial:**

International Standard Randomized Controlled Trial Number (ISRCTN): 15789289; http://www.controlled-trials.com/ISRCTN15789289 (Archived by WebCite at http://www.webcitation.org/6jGYZC3lZ)

## Introduction

Cancer-related information seeking behavior (ISB) is a goal-directed behavior adopted as a response to threatening situations, and it assists in uncertainty reduction concerning cancer [1]. Despite the importance of information seeking, the Health Information National Trends Survey has reported that less than half of Americans look for cancer information [2]. By promoting ISB [3], young adults may become aware of cancer risks and potential preventive measures. In particular, their active search for information about cancer may increase their cancer knowledge and equip them with ways to get protected from cancer [4,5].

One way to encourage ISB is by helping young adults perceive cancer risk [6]. Cancer risk perception is mainly characterized by two dimensions. First, perceived susceptibility to cancer explains one's beliefs about the likelihood of being diagnosed with cancer. The second dimension is perceived severity of cancer, which explains one's perception of the seriousness of cancer diagnosis. Previous research has found that young adults' perceived susceptibility to and severity of cancer may moderate engagement in healthy behaviors such as breast self-examination [7], mammography [8,9], skin protection [6], and smoking cessation [10].

Ultimately, the lack of ISB and cancer risk perception may delay cancer prevention and control at long term. As a result, the World Health Organization has emphasized the need to design interventions that successfully raise awareness about cancer risks and ISB [11]. While previous research has well examined processes by which individuals engage in health-related information seeking [12-14], little is known about the role of game play features in driving ISB. Responding to this need, video games have been designed as innovative tools for health promotion and disease prevention. The current study seeks to evaluate the impact of a video game called *“Re-Mission”* [15] on young adult college students' tendency to perceive the severity of cancer, feel susceptible to cancer, and seek cancer-related information.

*Re-Mission* is a fully automated game in which players control a virtual nanorobot that goes inside virtual cancer patients' bodies to fight cancer cells [16]. The *Re-Mission* intervention was designed primarily to encourage pediatric cancer patients to adhere to their medication [17]. However, recent exploratory research has shown that *Re-Mission* may have an impact on healthy young adults’ risk perception [18,19]. As a result, the evaluation of *Re-Mission* in the context of risk communication deserves attention. We conducted the current experimental evaluation [ISRCTN15789289] to verify whether and how *Re-Mission* might modify risk perception in healthy young adults. This would indicate whether there is a potential to design a digital game for the promotion of cancer preventing behaviors among young adults.

One theory supporting an association between game-play and cancer preventing behaviors is the protection motivation theory (PMT) [20,21]. According to PMT, threatening health messages can stimulate risk perception and encourage protective behavior. By experiencing threat, individuals may become motivated to take actions that can protect them, such as seeking information about the health topic [22-26]. Therefore, we hypothesized that young adults who play *Re-Mission* at ‘high challenge’ (HC) are more likely to increase perceived severity of cancer, perceived susceptibility to cancer, and cancer-related ISB, compared with young adults who play *Re-Mission* at ‘low challenge’ (LC) or do not play *Re-Mission*. We also hypothesized that (1) perceived threat in the gaming intervention is related to perceived susceptibility and perceived severity, and (2) such secondary outcomes are related to ISB.

## Methods

### Game Intervention Format and Key Features

The story in *Re-Mission* revolves around Roxxi, a nanorobot designed by a doctor and injected into the body of virtual cancer patients to help them fight cancer cells. Players are first presented with the narrative, the characters, and main game objective. Then, they are asked to choose a virtual cancer patient that needs assistance. The game gives the players control over the movement of Roxxi, who undertakes missions to fight cancer cells in a three-dimensional environment, within the bodies of cancer patients (See Multimedia Appendix 1).

Beyond mere exposure to information, *Re-Mission* involves a first-hand experience of cancer threat that ultimately allows players to perceive cancer risk. In *Re-Mission*, players are able to witness cancer cell behavior, from cell division to invasion, and ultimately find themselves in conflict with cancer cells. Conflict with cancer is a key feature of challenge in *Re-Mission*. A highly challenging environment with cancer cells (ie, presence of obstacles at high difficulty when fighting cancer cells) may facilitate perceived severity of cancer and intentions to seek cancer-related information [19].

### Study Design

The components of this controlled trial adhere to the CONSORT and CONSORT-EHEALTH guidelines [27,28] on information to include when reporting trials in general and eHealth in particular. This efficacy trial used a three-arm, single-blinded randomized controlled (Time × Condition) design with assessments at baseline, immediate posttest, 10-day, and 20-day follow-ups. The trial was registered at the Current Controlled Trials [ISRCTN15789289].

### Sample

We assessed the eligibility of all interested undergraduate students through a screening conducted before participation. Inclusionary criteria were being aged 18 to 35 years, attending college, consenting to play video games, and speaking English, Spanish, or French (the game was available in all three languages). Exclusionary criteria involved having a medical or mental condition that hindered the ability to play games or complete questionnaires. All participants were informed about the aim of the study and their consent for participation was recorded. The institutional review board at the University of Texas MD Anderson Cancer Center and the University at Buffalo, the State University of New York approved this study.

### Intervention Groups and Control Group

Young adults were randomly assigned to 1 of 3 conditions: an intervention group playing *Re-Mission* at HC (*n*=85), an intervention group playing *Re-Mission* at LC (n=81), and a control group with no challenge (NC; presented with illustrated pictures of *Re-Mission*; n=50).

Manipulation of challenge was conducted as suggested by previous research on conflict manipulation in *Re-Mission* [19]. HC was conceptualized as a condition that arises from a set of obstacles (eg, invasion by and multiplication of cancer cells) that prevent the players from attaining their goals in the game (eg, killing cancer cells and helping the patient recover from cancer) [29,30]. Three characteristics of challenge were manipulated in the game-mechanics to form an HC environment: (1) difficulty level (ie, amount of ammunition needed to destroy a cancer cell), (2) vulnerability to cancer cells (ie, cancer cells can put the nanorobot to sleep), and (3) limited ammunition (limited virtual medication to kill cancer cells). See Multimedia Appendix 2 for a pictorial depiction of manipulation.

The NC condition involved the presentation of several illustrations from the video game with a description of the conflict occurring between the nanorobot and the cancer cells. In particular, they were presented with illustrations of *Re-Mission* that represent steps of a conflict event with cancer cells (ie, exposure to cancer cells, cancer cells multiplying, Roxxi approaching cancer cells, cancer cells attacking Roxxi, and Roxxi fighting cancer cells; see examples in Multimedia Appendix 3). Each illustration included a textual description of the event and the context of the game. As a result, this condition lacked a first-hand experience of challenge, while keeping a presentation of the game available to participants. Instead of using a noneducational video game, the control condition of this study preserved the context of cancer prevention in order to highlight the role of conflict experience as a driver of health outcomes.

### Implementation

A verbal announcement was made in 3 undergraduate classes at a northeastern university. Each class included approximately 500 young adults. A Web-based announcement was also posted through the course material announcement page, and interested students were able to contact the research team for participation. Recruitment continued for a period of 2 months, or until reaching the target sample size. All interested students provided verbal and written informed consent and were invited for baseline assessment.

One week after completing a baseline survey, participants arrived at the intervention site and were randomly assigned to 1 of 3 conditions: LC, HC, or NC. The principal investigator generated the random allocation sequence. The research assistant enrolled participants and assigned them to groups. Concealed envelopes were used to implement the random allocation while concealing the sequence until intervention assignment. Participants were not told which intervention was the intervention of interest. Intervention implementation occurred in a noise-protected room at the university campus. Before playing *Re-Mission*, participants in the LC and HC conditions were seated in front of computers of the same brand and size, and they completed a tutorial that allowed them to practice using the controls in the game when attempting to move the avatar Roxxi (approximately 7 minutes). After the tutorial, all participants were seated in front of computers of the same brand and size, and were provided with headphones for privacy and maximum immersion. Then, participants were invited to start the first mission of the game and played for 35 minutes. Every time the players completed the mission and every time they lost in the game, they were asked to play it again, until the session was over. This method of intervention implementation with game interventions has been previously applied and validated with *Re-Mission* [19]. Participants in the NC group were also seated in front of computers, but they were presented with the NC condition of *Re-Mission*. For all conditions, the research assistant monitored progress from a different room.

Participants in all 3 conditions were invited to complete a survey immediately after implementation, 10 days later, and 20 days later. As an ethical consideration, after the 20-day follow-up, the NC group received information about *Re-Mission* and ways to access the game, if interested.

### Compensation

Participants were offered credits for their respective classes from their professors. Credits were provided for each assessment, and as a result of intervention participation (ie, 0.5 credit points for baseline survey, 1 credit point for intervention participation, 0.5 credit points for immediate post-test survey, and 1 credit point for each of the follow-up surveys).

### Measures

Outcome measures were assessed through Web-based closed surveys. The surveys were pretested for validity, reliability, usability, and technical functionality during the pilot study [19]. Adherence to the checklist for reporting results of Internet e-surveys [31] is provided as Multimedia Appendix 4. The post-test survey is the only survey that occurred in the presence of a research assistant, who was only available for technical assistance. The primary outcome ISB was assessed at baseline, 10-day, and 20-day follow-ups. The secondary outcomes, perceived severity of cancer and perceived susceptibility to cancer, were assessed at baseline, immediate posttest, 10-day, and 20-day follow-ups. In addition to the endpoints, other variables that might affect play behavior in a challenging virtual environment were measured (eg, frequency of weekly game play, perceived skills with video games, and perceived control over stress during game play). Perceived control over *Re-Mission* was measured to tap on players’ control over gaming events, including cancer cells during the challenge. Perceived threat from virtual cancer cells was measured at immediate posttest. Means, standard deviations, measure descriptions, and Cronbach’s α values are reported in Table 1. Previous work and the pilot study have tested the measures for validity and reliability [6,19,32-37].

**Table 1:** Main study measures.

Measures	T1^a^	T2^a^	Description	α^b^
	Mean (SD)	Mean (SD)		
ISB	2.12 (1.50)	2.16 (1.50)	Two items: “Have you paid attention to any cancer information in the past week or so?” and “Have you attempted to look for information about cancer in the past week or so?” (from 1=not at all to 9=a whole lot).	.71^d^	
Perceived susceptibility	2.92 (1.22)	3.17 (1.20)	Participants were asked how possible they were to contract cancer in the next year, in 5 years, in 10 years, and in their life-time (from 1=not at all possible to 7=extremely possible).	.91
Perceived severity	5.71 (1.28)	5.67 (1.13)	Four items such as “Cancer is a serious disease that can kill” (from 1=very strongly disagree to 7=very strongly agree).	.80
General control over stress	5.00 (0.98)	-	Ten items such as “I am able to control my level of anxiety while playing a video game” (from 1=very strongly disagree to 7=very strongly agree).	.79
General reaction to threat	4.64 (0.99)	-	Five items such as “There is little I can do to change threatening events” (Reverse coded; from 1=very strongly disagree to 7=very strongly agree).	.77
General perceived skills in game play	3.72 (1.34)	-	Six items such as “I am very skilled at playing shooting games” (from 1=very strongly disagree to 7=very strongly agree).	.85
Frequency of game play	2.71 (4.98)	-	One open-ended question: “How many hours per week do you spend playing computer games?”	-
Perceived control over *Re-Mission*	-	4.11 (1.84)	An adapted scale with 9 items such as “For me to feel in control over all cancer cells was difficult” (from 1=very strongly disagree to 7=very strongly agree).	.95
Perceived challenge	-	3.69 (1.39)	Four items such as “Playing *Re-Mission* has challenged me to perform to the best of my abilities” (from 1=very strongly disagree to 7=very strongly agree).	.90
Perceived threat from virtual cancer cells	-	4.70 (2.27)	Four 9-point semantic differential items such as “While playing *Re-Mission*, how threatening did you feel cancer cells to be?” (from 0=not at all threatening to 8=extremely threatening).	.92
Attitude toward *Re-Mission*	-	4.22 (1.70)	Eight 9-point semantic differential items (e.g., dislike/like and not worth owning/worth owning).	.91	

^a^T1 and T2 indicate measures at pretest and post-game play respectively for all participants. T2 for ISB indicates 20-day follow-up.

^b^Coefficients for Cronbach’s α were calculated from post-test data, with the exception for measures with data collected at T1 only.

^c^Standard deviations appear in parentheses below the mean.

^d^Indicates Pearson’s correlation between 2 items, instead of Cronbach’s α.

### Sample Size and Statistical Analysis

Sample size was estimated on the basis of a previous pilot study of 44 young adults [19]. Analyses targeted detection of an effect size of 0.15 (Cohen's d) with 85% power and α=0.05 (two-sided), with adjustment for an anticipated 70.0% (n=102/145) retention rate.

Statistical analyses were conducted using STATA 12. First, a series of chi-squared analyses were conducted to check for any sociodemographic differences between the groups. Then, manipulation checks were conducted in order to check if the manipulation appropriately reflects levels of challenge. This involved a series of one-way analyses of variance (ANOVAs), checking for group differences with respect to prior gaming experience (ie, skills in digital game play, general reaction to threat during game play, control over stress, or prior history of game play in hours per week). The ANOVAs also checked for any group difference in attitude toward *Re-Mission*, perceived control over game play, and experience of positive challenge during game play. When warranted, Bonferroni adjustment was performed, correcting for alpha over repeated comparisons and guarding against Type 1 error [38].

Repeated-measures, mixed-effect linear models were used, testing differences between the three treatment groups at three time-points in a 3 (treatment) × 3 (time; baseline, 10-day and 20-day follow-up) factorial design for ISB, and at four time-points in a 3 (treatment) × 4 (time; baseline, post-test, 10-day, and 20-day follow-up) factorial design for perceived cancer severity and susceptibility. ISB was not measured at immediate posttest because participants did not yet have the chance to seek cancer information. Intervention effects on the change in outcomes over time were determined by the treatment × time interaction term and *P* values are reported. Change over time is analyzed using post-hoc tests of significant difference in scores between time points, and *P* values are reported.

One-way ANOVA was conducted to compare the groups with respect to ISB change. ISB change is a variable measured by subtracting the ISB score at baseline from the ISB score at 10-day follow-up.

Logistic regression analysis was also conducted to determine group differences in ISB at three time-points (baseline, 10-day, and 20-day follow-up). In this case, the ISB measure was treated as a dichotomous variable with “not at all” indicating no information seeking (coded 0), and all other answer choices indicating information seeking (coded 1). Results were determined with the odds ratio (OR) and 95% confidence interval (CI).

For mixed-effect and logistic regression models, adjustment for effects of gender, age, ethnic group, prior cancer history, gaming skills, and usual frequency of gameplay did not alter primary conclusions (analyses not shown).

To check for potential demographic confounders, we analyzed mixed-effect models with the interactions (1) gender × condition × time, (2) ethnicity × condition × time, (3) prior cancer history × condition × time, and (4) general perceived skills in game play × condition × time predicting ISB.

To test whether perceived threat in the gaming experience is related to the secondary outcomes (ie, perceived susceptibility and perceived severity), two repeated-measures, mixed-effect models were conducted controlling for the intervention effect, age, gender, and ethnicity. Also, to test whether perceived susceptibility and perceived severity are related to the primary outcome (ie, ISB), a repeated-measures, mixed-effect model was conducted controlling for the intervention effect, age, gender, and ethnicity.

## Results

### Attrition and Intervention Adherence

**Figure 1:**
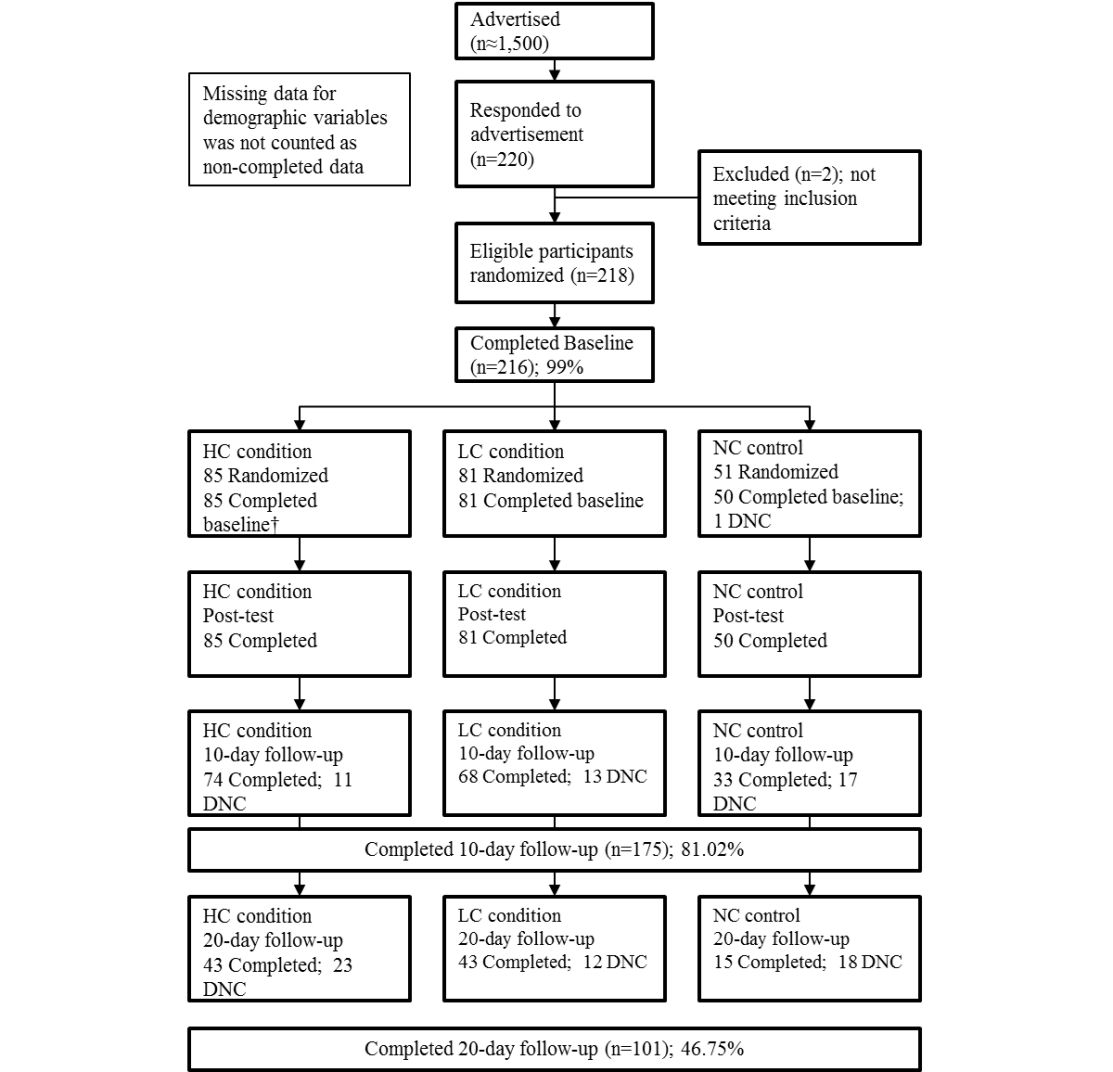
CONSORT flow diagram. DNC, participants who did not continue in the study.

A total of 220 college students responded to the advertisement. After screening, we excluded 2 of the respondents who did not meet the young-adult age criterion (ages 18 through 35). A total of 216 young adults took the baseline survey, were randomized, participated in the intervention, and completed a post-test survey. At the intervention site, all participants played *Re-Mission* as prescribed. Then, 81.02% (175/216) of participants continued to 10-day follow-up (retention rate from baseline), and 46.76% (101/216) continued to 20-day follow-up (Figure 1).

There was no significant difference between participants who did and those who did not continue to 10-day follow up assessment with respect to age (F_1,201_=3.40, *P*=.07), gender (χ^2^_1_=0.17, *P*=.68), ethnicity (χ^2^_4_=7.08, *P*=.13), usual frequency of gameplay at preintervention (F_1,203_=0.09, *P*=.76), or control over stress (F_1,180_=0.02, *P*=.90). Similarly, there were no differences between participants who did and those who did not continue to 20-day follow-up with respect to age (F_1,201_=0.08, *P*=.78), gender (χ^2^_1_=0.03, *P*=.85), ethnicity (χ^2^_4_=4.65, *P*=.32), usual frequency of gameplay at preintervention (F_1,203_=0.07, *P*=.79), or control over stress (F_1,180_=0.17, *P*=.68). Sociodemographic characteristics of the 3 groups did not differ significantly at baseline (Table 2).

**Table 2:** Baseline participants’ characteristics.

Characteristics		Participants, n (%)^a^				*P* ^b^
		No challenge (control) (n=50)	Low challenge (n=81)	High challenge (n=85)	Total sample (n=216)	
**Age, years**						
	18	3 (6.0)	20 (24.7)	15 (17.7)	38 (17.6)	.1
	19	10 (20.0)	21 (25.9)	21 (24.7)	52 (24.1)	
	20	12 (24.0)	13 (16.1)	24 (28.2)	49 (22.7)	
	21	9 (18.0)	9 (11.1)	13 (15.3)	31 (14.6)	
	≥22	12 (24.0)	11 (13.6)	9 (10.6)	32 (14.8)	
	Missing	4 (8.0)	7 (8.6)	3 (3.5)	14 (6.5)	
**Gender**						
	Male	19 (37.3)	37 (45.7)	43 (50.6)	99 (45.6)	.5
	Female	27 (54.0)	37 (45.7)	39 (45.9)	104 (47.9)	
	Missing	4 (8.0)	7 (8.6)	3 (3.5)	14 (6.5)	
**Race/ethnicity**						
	White/Caucasian	34 (68.0)	50 (61.7)	46 (54.1)	131 (60.2)	.1
	Asian	4 (8.0)	15 (18.5)	21 (24.7)	40 (18.5)	
	Hispanic/Latino	1 (2.0)	4 (4.9)	3 (3.5)	8 (3.7)	
	African American	4 (8.0)	3 (3.7)	10 (11.8)	17 (7.9)	
	American Indian/Alaska Native	2 (4.0)	0 (0.0)	1 (1.12)	3 (1.4)	
	Missing	5 (10.0)	9 (11.1)	4 (4.7)	18 (8.3)	
**Prior cancer screening**						
	Yes	8 (15.7)	13 (16.1)	11 (12.9)	32 (14.8)	.7
	No	38 (74.5)	61 (75.3)	70 (82.4)	169 (77.9)	
	Missing	5 (9.8)	7 (8.6)	4 (4.7)	16 (7.4)	
**Prior cancer diagnosis**						
	Yes	1 (2.0)	1 (1.2)	0 (0.0)	2 (14.8)	.6
	No	46 (90.2)	73 (74.1)	81 (85.9)	200 (79.6)	
	Missing	4 (7.8)	7 (8.6)	4 (4.7)	15 (6.9)	
						

^a^Percentages may not sum to 100% due to rounding.

^b^Test of association was done from chi-squared test (categorical variables), excluding categories of missing values. No differences were found in demographic characteristics between groups.

**Table 3:** Manipulation checks to confirm expected differences and similarities between the conditions.

Variables		Control	Low challenge	High challenge	*F* ^a^	*P*	η^2^
		Mean (SD)	Mean (SD)	Mean (SD)			
**Manipulation-dependent personality traits^b^ and gaming experience**							
	General perceived skills in game play	3.77 (1.26)	3.72 (1.37)	3.71 (1.36)	0.01	.98	<.001
	General reaction to threat	4.47 (1.00)	4.64 (0.98)	4.70 (1.04)	0.45	.63	.005
	General control over stress	4.93 (0.94)	4.98 (1.05)	5.04 (0.94)	0.14	.87	.001
	Number of hours of game play per week	3.00 (5.31)	1.70 (3.13)	2.79 (8.50)	0.83	.44	.008
**Expected manipulation outcomes^c^**							
	Attitude toward *Re-Mission*	3.96 (1.61)	4.32(1.65)	4.28 (1.80)	0.74	.48	.006
	Perceived control over *Re-Mission*	-	4.42 (1.55)	3.29 (1.21)	27.39	<.001	.14
	Perceived challenge in playing *Re-Mission*	-	3.33 (1.47)	4.04 (1.22)	12.06	<.001	.07
	Perceived threat from cancer cells	-	4.21 (2.22)	5.18 (2.19)	8.02	.005	.05

^a^Eight separate one-way ANOVAs analyzing the differences between the conditions.

^b^Personality traits that are considered are those that may affect the manipulation of challenge. No differences were found in such traits between groups.

^c^Outcomes that may be affected by the manipulation of challenge. Variables that are specific to *Re-Mission* were not considered in the control group.

### Manipulation Checks

A series of ANOVAs were conducted to check for any differences in prior game experience between the conditions, as well as any personality traits that may affect the manipulation (Table 3). There were no significant differences between the conditions with regard to skills in digital game play, general reaction to threat during game play, control over stress when playing games, or prior history of game play.

After the intervention, there was no significant difference between the conditions with respect to attitude toward *Re-Mission*. As expected, players in the HC condition, compared with the LC condition, were less likely to perceive control over game play. In addition, players at HC were more likely to experience positive challenge and perceive threat from cancer cells in the game (Table 3).

At baseline, there were no significant differences between the conditions with respect to perceived severity (F_2,110_=0.49, *P*=.61) or ISB (F_2,139_=0.49, *P*=.17). However, perceived susceptibility to cancer was greater among participants in the control group, compared with participants in the HC group (F_2,110_=3.46, *P*=.03). Subsequent analyses controlled for this baseline group difference by including the baseline variable as an independent variable in the mixed-effect model.

### Checking for Confounders

To check for potential demographic confounders, we determined whether intervention effects varied by gender, ethnicity, prior cancer history, or perceived gaming skills. Results generally failed to identify any differential impact as a function of being female (χ^2^_2_=1.51, *P*=.47), being White/Caucasian (χ^2^_2_=5.25, *P*=.07), having personal or social cancer history (χ^2^_2_=1.04, *P*=.59), or reporting having skills in game play (F_2,176_=0.01, *P*=.98).

### Insuring Power for Main Data Analysis

Post-hoc power analysis for a repeated-measures analysis to test perceived susceptibility or perceived severity revealed that with 3 groups, 4 repeated measures, a constant correlation of 0.5, an alpha of 0.05, and a sample size of 101, there was 90.70% power to detect small-to-moderate overall effect (Cohen's d=0.15). The same power analysis to test ISB revealed that with 3 groups, 3 repeated measures, a constant correlation of 0.5, an alpha of 0.05, and a sample size of 101, there was 84.66% power to detect small-to-moderate overall effect (Cohen's d=0.15).

### Perceived Susceptibility to Cancer

**Figure 2:**
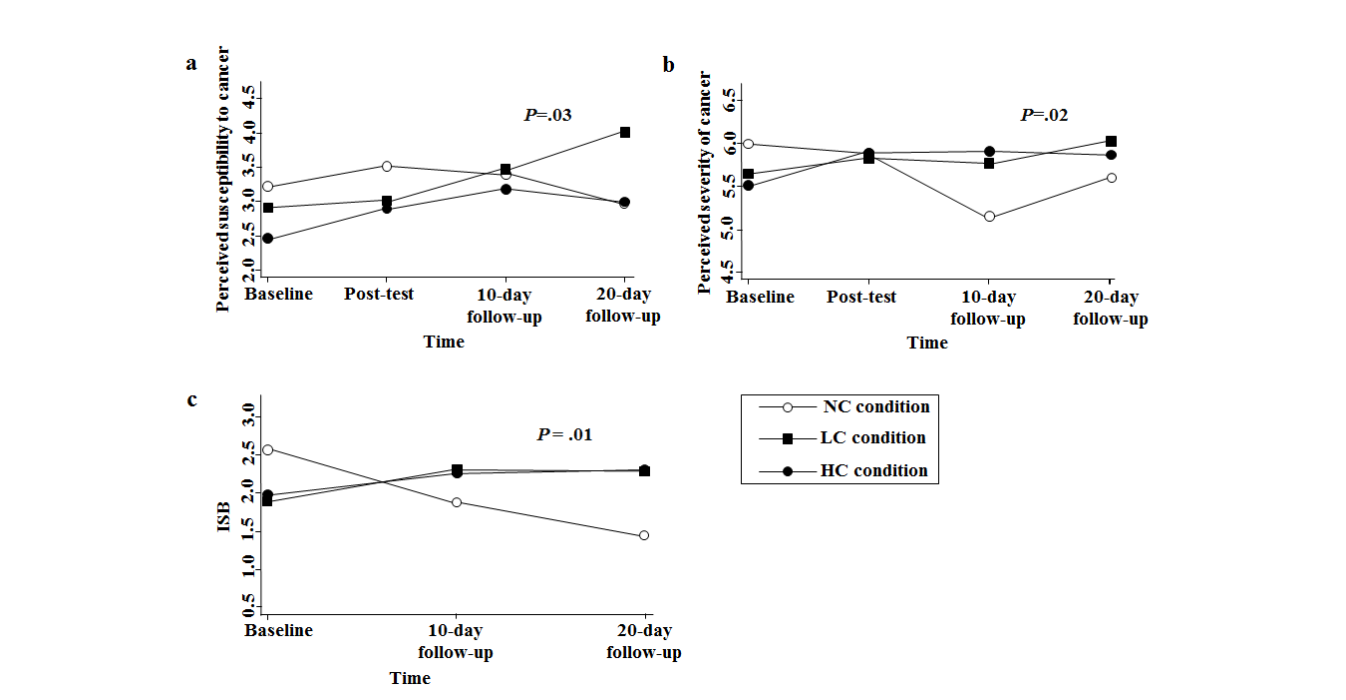
Adjusted predictions of condition-by-time. ISB, information seeking behavior; NC, no challenge; LC, low challenge; HC, high challenge. P-values present significance for the group × time interaction effect.

Mixed-effect models showed a significant group × time interaction effect on perceived susceptibility to cancer (*P*=.03; Figure 2 a). For the LC group, there was no significant increase in perceived susceptibility from baseline to posttest (B=0.09, SE 0.16, *P*=.56). However, a significant increase was observed from posttest to 10-day follow-up (B=0.47, SE 0.16, *P*=.005) and a marginal increase from 10-day to 20-day follow-up (B=0.56, SE 0.31, *P*=.07). For the HC group, a significant increase in perceived susceptibility was observed from baseline to posttest (B=0.43, SE 0.14, *P*=.002), which then exhibited a plateau with no significant change from posttest to 10-day follow-up (B=0.30, SE 0.18, *P*=.10) and from 10-day to 20-day follow-up (B=−0.19, SE 0.26, *P*=.46). For the NC control group, no significant change was observed from baseline to 20-day follow-up (B=−0.26, SE 0.38, *P*=.50; Figure 2 a).

### Perceived Severity of Cancer

A significant group × time interaction effect is also observed for perceived severity (*P*=.02; Figure 2 b). The LC group exhibited no significant changes from baseline to 20-day follow-up (B=0.40, SE 0.33, *P*=.24). On the other hand, the HC group showed a significant increase from baseline to posttest (B=0.39, SE 0.14, *P*=.005), which plateaued from posttest to 10-day follow-up (B=0.005, SE 0.18, *P*=.98) and 10-day to 20-day follow-up (B=−0.007, SE 0.26, *P*=.98). For the NC control group, no significant change is observed from baseline to posttest (B=−0.13, SE 0.18, *P*=.48). However, from posttest to 10-day follow-up, the NC group showed a significant decrease in perceived severity (B=−0.74, SE 0.24, *P*=.002), with no significant change from 10-day to 20-day follow-up (B=0.46, SE 0.32, *P*=.15; Figure 2 b).

### Information Seeking Behavior

Mixed-effect analysis showed a significant group × time interaction effect on cancer information seeking by 20-day follow-up (*P*=.01; Figure 2 c). From baseline to 10-day follow-up, participants in the LC group (B=1.09, SE 0.40, *P*=.006) and the HC group (B=0.97, SE 0.38, *P*=.01) were more likely to increase in ISB, compared with participants in the NC group. Also, from baseline to 20-day follow-up, participants in the LC group (B=1.50, SE 0.52, *P*=.004) and the HC group (B=1.42, SE 0.51, *P*=.005) were more likely to increase in ISB, compared with participants in the NC group. Interestingly, ISB significantly decreased for NC from baseline to 20-day follow-up (B=−1.11, SE 0.43, *P*=.01). One-way ANOVA indicated that change in ISB over time (ISB at 20-day follow up – ISB at baseline) was significantly higher for LC compared with NC (F_2,42_=3.60, *P*=.004), and higher for HC compared with NC (F_2,42_= 2.96, *P*=.008).

With ISB as a dichotomous variable, mixed-effect logistic regression indicated a significant group × time interaction effect (*P*=.007). Participants in the LC group were more likely to have sought cancer information at 10-day follow-up (OR 5.10, 95% CI 1.06-24.46, *P*=.04) and 20-day follow-up (OR 121.89, 95% CI 7.05-2105.88, *P*=.001), compared with participants in the NC group. This relationship was also significant for HC compared with NC at 10-day follow-up (OR 6.26, 95% CI 1.40-28.11, *P*=.02) and 20-day follow-up (OR 107.23, 95% CI 7.17-1602.69, *P*=.001).

### Relationship Results

Mixed-effect analysis revealed that perceived threat from the virtual cancer cells was significantly related to an increase in perceived severity of cancer (B=0.1, SE 0.03, *P*=.001). However, there was no significant relationship between perceived threat and perceived susceptibility to cancer (B<0.001, SE 0.03, *P*=1.00).

Mixed-effect results indicated that increases in perceived susceptibility were significantly related to increases in ISB from baseline to 10-day follow-up (B=0.21, SE 0.08, *P*=.008). There was a marginal significant relationship between the increase in perceived susceptibility and the increase in ISB by 20-day follow-up (B=0.16, SE 0.09, *P*=.09). On the other hand, increases in perceived severity were not related to increases in ISB by 10-day follow-up (B=−0.07, SE 0.08, *P*=.40), or 20-day follow-up (B=−0.001, SE 0.09, *P*=.99). When considering perceived susceptibility and severity in the same model, perceived susceptibility still exhibits a significant relationship with ISB (B=0.21, SE 0.08, *P*=.007), while perceived severity exhibits no relationship with ISB (B=−0.08, SE 0.08, *P*=.33).

## Discussion

### Conclusions

While *Re-Mission* has shown success in promoting medication adherence in young cancer patients [17], this randomized controlled study showed that the experience of challenge in *Re-Mission*, when played by young healthy adults, led to an increase in perceived cancer severity and susceptibility, as well as a seeking of cancer-related information. The findings also indicated that the perception of threat during the intervention increased perceived severity of cancer.

This trial is the first to present the potential use of *Re-Mission* for cancer risk communication among healthy young adults. Gaming features in *Re-Mission* increased young adults’ cancer risk perception and led to the seeking of cancer-related information. The critical gaming feature manipulated in this study is challenge, which in *Re-Mission* is represented as the conflict between young-adult players and cancer cells. This conflict is characterized by the proliferation of cancer cells and their continuous attack of the player avatar, Roxxi [19].

The results indicate that a high level of challenge in the intervention led to a quick (posttest) change in perceived susceptibility, whereas a low level of challenge was associated with a slower change (10-day follow-up), which marginally increased in the longer term (20-day follow-up). This indicates that the level of challenge may not need to be high to promote enduring change in perceived susceptibility.

Further, the results suggest that a high level of challenge may be needed to change perceived severity of cancer and to maintain that change in the longer term. The HC group exhibited an increase from baseline to posttest, which then plateaued from posttest until 20-day follow-up. On the other hand, the LC group showed no changes from baseline to posttest, 10-day or 20-day follow-up. As hypothesized previously [19], the HC condition, in comparison to the LC condition, exposes players to more aggressive behavior in cancer cells, an experience likely to lead them to perceive cancer as more severe.

The results highlight the limits of mere exposure to health information as opposed to virtual experience of challenge by cancer cells. For the NC control group, no change in perceived severity or perceived susceptibility is observed from baseline to posttest. However, from posttest to 10-day follow-up, the NC group showed a significant decrease in perceived severity. Following the presentation of information that describes cancer cell behavior, young adults did not display any increase in their perception of the severity of cancer, and even showed a decrease in the longer term. Such results are consistent with other research indicating that interactive experience is an important determinant of perceived severity of cancer [39,40].

There was no significant difference between the HC and LC groups in ISB variation over time (Figure 2 c). However, compared with the NC group, the HC and LC groups were more likely to demonstrate an increase in ISB from baseline to 10-day and 20-day follow-up. For this reason, the difference in ISB change between the NC group and the other groups may be driven by factors other than perceived severity.

Our relationship results also attest to this finding. Perceived threat, a direct outcome of the intervention, was related to changes in perceived severity but not susceptibility. However, it is perceived susceptibility that was found to be related to ISB. As a result, the antecedents of perceived susceptibility warrant further investigation in order to understand how ISB occurs following the gaming intervention.

Our previous investigations with *Re-Mission* indicated that threat perception is associated with fear when facing the cancer cells in the game [41]. Effects of perceived threat can be further investigated during future research, in order to understand the role of emotions in driving health outcomes.

### Limitations

In this study, young adults participated in 1 session of *Re-Mission* only. Typical use of *Re-Mission* might involve many hours of play over a period of time, and the results found in this study might not be characteristic of more extensive play. The results may also be affected by the tutorial, which can be skipped in a typical setting. However, all *Re-Mission* players were presented with the tutorial to control for its potential effect.

This study ended with a relatively low retention rate (101/216, 46.8%). By the time this study reached 20-day follow-up, college students were at a transition to summer break, and ultimately, several of them were not available to continue in the study. However, this did not stop 101 participants to continue in the study, and keep acceptable power for data analysis. Future work with college students may need to consider a more suitable timing for data collection.

While the results explain short-term effects on information seeking, they do not consider long-term opportunities for actual protective behaviors. Notably, though, this preclinical trial was meant only to test the potential effectiveness of challenge as a moderator of risk perception and ISB. The current study did not inspect specific types of ISB. However, our pilot study indicated relationships between general ISB and players’ intentions to obtain information from family members and from doctors during medical visits [19].

*Re-Mission* is not designed to directly influence actual cancer preventive behaviors among healthy young adults (eg, improvement in healthy eating or prevention of tobacco smoking). However, our results indicate that conflict with cancer cells and the virtual experience of cancer cell behavior could increase young-adults’ comprehensive understanding and perception of cancer, which could well act as a driver for acquisition of preventive behaviors.

### Implications

The results of this study can be used to inform the design of a novel, game-based intervention for cancer risk communication. Such an intervention might make use of the current findings by providing a balance between LC and HC during conflict with virtual cancer cells. With challenge manipulation, the intervention may create a state of balance between the level of challenge in the game and players’ ability to overcome the challenge. Also, the new intervention could allow young adults to discover new cancer information inside the game, and ultimately learn about new ways to protect themselves. With cancer information embedded in the intervention, it may become possible to measure ISB objectively by monitoring the player’s accessing of information in the game. The new intervention may boost self-efficacy for self-protection by allowing young adults to enter a virtual environment that facilitates the simulation of engagement in healthy actions. While challenge with cancer cells may drive risk perception, it can also allow young adults to experience the consequences of their actions in the game. In particular, a new intervention may allow young adults to explore the risks of cancer-promoting behaviors (eg, smoking), as well as the benefits of cancer preventive behaviors (eg, healthy eating). Finally, with management options in the game, young adults can create a plan and register electronic reminders to engage in preventive behaviors. For instance, through the game, college students can make Web-based appointments for cancer screening or vaccination at the clinics of their Universities.

## Appendix

Multimedia Appendix 1 A video of the *Re-Mission* game presenting the conflict between Roxxi and the cancer cells.

Multimedia Appendix 2 Screenshots of the *Re-Mission* settings interface that allow the manipulation of the features of challenge.

Multimedia Appendix 3 Screenshot of the layout and design of *Re-Mission*; an illustration from the “no challenge” (NC) condition.

Multimedia Appendix 4 Checklist for reporting results of Internet e-surveys (CHERRIES).

## Abbreviations

ANOVA: analysis of the variance
CI: confidence interval
HC: high challenge
ISB: information seeking behavior
LC: low challenge
NC: no challenge
OR: odds ratio
PMT: protection motivation theory
SE: standard error
