# Supplementary figures and images for: A Video Game Promoting Cancer Risk Perception and Information Seeking Behavior Among Young-Adult College Students: A Randomized Controlled Trial
Source: JMIR Serious Games. 2016 Jul 28;4(2):e13. doi: 10.2196/games.5793 (PMC4981692; doi:10.2196/games.5793)

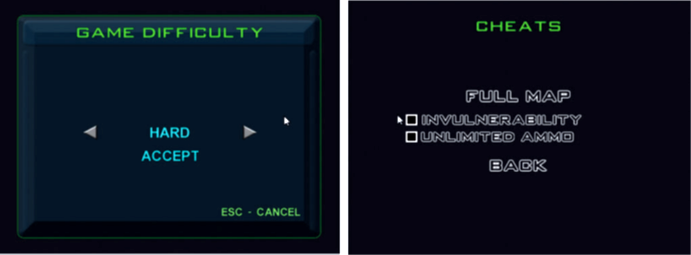

Supplement: Multimedia Appendix 2 [file games_v4i2e13_app2.png]

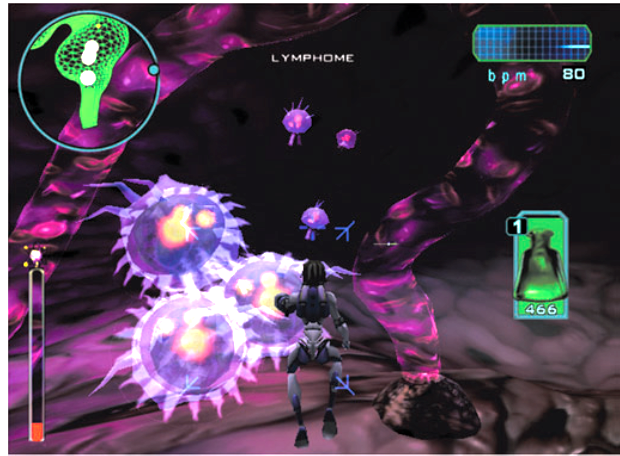

Supplement: Multimedia Appendix 3 [file games_v4i2e13_app3.png]
