# Supplementary material for: A Video Game Promoting Cancer Risk Perception and Information Seeking Behavior Among Young-Adult College Students: A Randomized Controlled Trial
Source: JMIR Serious Games. 2016 Jul 28;4(2):e13. doi: 10.2196/games.5793 (PMC4981692; doi:10.2196/games.5793)
Supplement: Multimedia Appendix 4 [file games_v4i2e13_app4.pdf]

Checklist for Reporting Results of Internet E-Surveys (CHERRIES)

Design

The e-surveys of the present study were aimed to collect data from young adult college students. The surveys were administered at baseline, post-test (after game play), 10-day follow-up, and 20-day follow-up.

Development and Pre-testing

All the surveys were designed using SurveyMonkey [1]. The measures were chosen based on previous research in risk communication and game play for persuasion [2-7]. The surveys were also pre-tested for usability and technical functionality during the pilot study [5]. During the pilot study, participants were encouraged to ask questions concerning the surveys and report any concerns to the research team. The short scale of perceived threat from virtual cancer cells was found to be too brief, and as a result, it was improved in the current trial by including additional items that tap on threat perception.

Forest-Boucher, Brunet, and Fredette [2] as well as Novak and Hoffman [7] have tested the validity and reliability of perceived skills in game play and perceived challenge during game play (Cronbach's alphas between 0.87 and 0.94). Factor analysis showed separate factors for the concepts and predictive validity indicated that such concepts predict the experience of flow during game play (i.e., balance between difficulty of game and ability to overcome challenge in the game) [2, 7]. Rapee and colleagues [4] tested the validity and reliability of perceived control over stress (Cronbach's alpha=0.89). Kraft and colleagues [6] tested the validity and reliability of a measure for perceived control over the game (Cronbach's alpha=0.77).

The pilot study [5] reiterated the validity and reliability of these four measures in the context of the video game "Re-Mission". Factor analysis indicated separate factors for such measures and they correlated with perceived difficulty of the game. The pilot study also indicated good internal reliability of the measures: perceived skills in game play (Cronbach's alpha=0.90), perceived challenge (Cronbach's alpha=0.93), perceived control over stress (Cronbach's alpha=0.90), and perceived control over the game (Cronbach's alpha=0.96).

Perceived severity of cancer has been tested by Rimal and Real [3], and perceived susceptibility to cancer has been adapted from the work of Rimal and Real [3]. In addition, the pilot study has shown the validity and reliability of the two scales in measuring perceived susceptibility and severity (Cronbach's alphas between 0.79 and 0.91). During the pilot study, validity indicated that perceived threat from cancer cells predicted perceived susceptibility to cancer (β=0.37, $p<0.05$) and perceived severity (β=0.32, $p<0.05$).

Finally, information seeking behavior (ISB) is a measure adapted from the information seeking measure of Rimal and Real [3]. It taps on respondents' general information-seeking tendency. During the pilot study, the 2-item scale showed internal consistency with a significant correlation between the two items ($r=0.85$, $p<0.0001$) and a Cronbach's alpha of 0.91. Also, during the pilot study, predictive validity showed that the measure predicted social information seeking through general discussion of cancer with family ($r=0.35$, $p<0.05$), with friends ($r=0.43$, $p<0.001$), and during doctor's visits ($r=0.41$, $p<0.001$).

IRB Approval, Informed Consent Process, Recruitment, and Survey Administration

The study involving these surveys has been approved by the institutional review board.

The initial contact with potential participants was made face-to-face through an announcement. One month before the experiment, the research team attended classes at the University at Buffalo, to announce the study and invite participation. The announcement reached as many as 500 students.

In addition to the verbal announcement, an announcement was posted online through the class websites. The class website can only be visited by the young adult students attending the class. The website already pre-selects this group, which forms the population of interest.

The online and the face-to-face announcements explained the purpose of the study, the length of the study, method of accessing the study sign-up sheet, risks/benefits of the study, and statement of voluntary participation. The announcements included a link to the baseline survey.

The baseline survey began with an electronic consent form specific to the survey. The baseline survey was closed to the subsample of young adult students who were interested in the study and who have approved the electronic consent form. This consent form was also provided in hard copy for the students to keep for their own records, after they arrived at the experimental site. At the experimental site, participants were provided with an informed consent form for the rest of the study, which was verbally explained. All other surveys were also closed surveys that were only open for participants who continued in the study.

For all the surveys, no personal information was collected, except for e-mail addresses. However, all data was retrieved from the cloud and stored in a password protected hard drive and locked in a data storage room. Only the principal investigator had access to the data.

Participation in the study was voluntary, and participants were not required to complete the surveys. Participants were compensated by receiving additional course credits for completing the surveys and taking part in the intervention.

The baseline survey was open for a period of two months. The post-test survey was taken at the intervention site immediately after game play. The 10-day and 20-day follow-up surveys were sent to each participant via e-mail. Each survey had between 1 and 13 items per page. The surveys did not allow for the participants to review answers. The participants could not go to a previous page to change their answers.

Response Rate

Considering the need for full privacy, IP addresses were not collected for participants. Instead, a unique participant was determined based on the university e-mail address they provide on the first page of the survey. The participation rate for all surveys was 100%. The completion rate for the baseline survey was 99%. The completion rate for the post-test survey was 100%. The completion rate for the 10-day follow-up survey was 81.02%. The completion rate for the 20-day follow-up survey was 57.71%.

Preventing Multiple Entries

While the surveys were closed, they did not involve a login to prevent duplicate entries. For all surveys, IP addresses were not collected based on the requirements of the

institutional review board. However, duplicate database entries having the same user ID were eliminated before analysis, such that the first entry was kept and the second entry was eliminated.

Analysis

All the data including incomplete questionnaires were analyzed. All participants provided answers until the last page of the surveys. No statistical method was needed to correct for missing values.

References

1. Gordon A. SurveyMonkey. com—Web-Based Survey and Evaluation System: http://www.SurveyMonkey.com, The Internet and Higher Education 2002: 5: 83-87.
2. Forest-Boucher A, Brunet J, Fredette M. Brand placement in virtual environments: Impact of flow on brand recognition and recall. Les Cahiers du Gerad 2008;75:1-22. ISSN:0711–2440
3. Rimal RN, Real K. Perceived risk and efficacy beliefs as motivators of change. Hum Commun Res 2003;29(3):370-399. DOI: 10.1093/hcr/29.3.370
4. Rapee RM, Craske MG, Brown TA, Barlow DH. Measurement of perceived control over anxiety-related events. Behav Ther 1996;27(2):279-293. PMID:12102581
5. Khalil GE. When losing means winning: The impact of conflict in a digital game on young adults' intentions to get protected from cancer. Games Health J 2012;1(4):279-286. PMID:26191631
6. Kraft P, Rise J, Sutton S, Røysamb E. Perceived difficulty in the theory of planned behaviour: Perceived behavioural control or affective attitude? Brit J Soc Psychol 2005;44(3):479-496. PMID:16238850
7. Novak TP, Hoffman DL. Measuring the flow experience among web users. Proceedings of the Interval Research Corporation; 1997 Jul 31; Nashville, Tennessee. USA: Project 2000, Vanderbilt University.
